# Diagnosis of COVID-19 Pneumonia Based on Graph Convolutional Network

**DOI:** 10.3389/fmed.2020.612962

**Published:** 2021-01-21

**Authors:** Xiaoling Liang, Yuexin Zhang, Jiahong Wang, Qing Ye, Yanhong Liu, Jinwu Tong

**Affiliations:** ^1^Department of Marine Engineering, Dalian Maritime University, Dalian, China; ^2^Department of Electrical and Computer Engineering, National University of Singapore, Singapore, Singapore; ^3^School of Instrument Science and Engineering, Southeast University, Nanjing, China; ^4^Department of Electrical and Computer Engineering, University of Illinois at Urbana-Champaign, Champaign, IL, United States; ^5^Division of Life Sciences and Medicine, Department of Pathology, The First Affiliated Hospital of USTC, University of Science and Technology of China, Hefei, China; ^6^Division of Life Sciences and Medicine, Intelligent Pathology Institute, University of Science and Technology of China, Hefei, China; ^7^Department of Pathology, Nanjing Drum Tower Hospital, The Affiliated Hospital of Nanjing University Medical School, Nanjing, China; ^8^School of Innovation and Entrepreneurship, Nanjing Institute of Technology, Nanjing, China

**Keywords:** COVID-19, graph convolutional network, 3D convolutional neural network, equipment types, chest computed tomography

## Abstract

A three-dimensional (3D) deep learning method is proposed, which enables the rapid diagnosis of coronavirus disease 2019 (COVID-19) and thus significantly reduces the burden on radiologists and physicians. Inspired by the fact that the current chest computed tomography (CT) datasets are diversified in equipment types, we propose a COVID-19 graph in a graph convolutional network (GCN) to incorporate multiple datasets that differentiate the COVID-19 infected cases from normal controls. Specifically, we first apply a 3D convolutional neural network (3D-CNN) to extract image features from the initial 3D-CT images. In this part, a transfer learning method is proposed to improve the performance, which uses the task of predicting equipment type to initialize the parameters of the 3D-CNN structure. Second, we design a COVID-19 graph in GCN based on the extracted features. The graph divides all samples into several clusters, and samples with the same equipment type compose a cluster. Then we establish edge connections between samples in the same cluster. To compute accurate edge weights, we propose to combine the correlation distance of the extracted features and the score differences of subjects from the 3D-CNN structure. Lastly, by inputting the COVID-19 graph into GCN, we obtain the final diagnosis results. In experiments, the dataset contains 399 COVID-19 infected cases, and 400 normal controls from six equipment types. Experimental results show that the accuracy, sensitivity, and specificity of our method reach 98.5%, 99.9%, and 97%, respectively.

## Introduction

The first case of COVID-19 was described in China in December 2019, and then COVID-19 has spread all over the world rapidly. So far, it has infected over 31 million people and has resulted in over 0.9 million deaths as of September 23, 2020. With the numerous cases needed to be tested, most countries and regions face a shortage of testing kits and medical resources. For this issue, a series of automatic diagnosis methods based on deep learning models are proposed to relieve the medical burden (1). Unlike the reverse-transcription polymerase chain reaction (RT-PCR) based on a patient's respiratory samples, automatic diagnosis methods based on deep learning models usually accomplish the diagnosis task by using chest radiography images (2). Various published research articles indicate that chest scans are useful in detecting COVID-19 (3, 4). The lungs of the infected cases have visual marks like ground-glass opacity or hazy darkened spots, which help to differentiate infected cases from normal controls (5). With good sensitivity (SEN) and speed, chest computed tomography (CT) has been widely used in automatic diagnosis methods (6–9).

For the existing automatic diagnosis methods of COVID-19 based on medical images, structures based on a convolutional neural network (CNN) are widely used. For example, the DarkNet model (10) with 17 convolutional layers is used as a classifier for COVID-19 diagnosis based on X-ray images, and its accuracy (ACC) reaches 98.08%. A transfer learning neural network (11) based on the inception network is proposed to fit in few-shot CT datasets, and it achieved a total ACC of 89.5%. A deep three-dimensional (3D)-CNN (12) is applied to detect COVID-19 from CT volumes reaching 90.8% ACC. Additionally, ResNet50 (13), ResNet152 (14), LSTM (15), GAN (16, 17), and some other structures are successively used for COVID-19 diagnosis. Limited by the insufficient training samples and the great number of parameters in deep learning structures, the ACC of the above methods based on 3D-CT images is not satisfied. Besides, in most of the existing automatic diagnosis methods, the differences between image standards from different equipment types and hospitals are ignored, which further deteriorates the final diagnosis performance. As COVID-19 widely spreads, it is of great significance to exploit a robust diagnosis method to adapt to different acquired equipment types all over the world.

A graph convolutional network (GCN), which integrates phenotypic information into a graph to establish interactions between individuals and populations, can achieve an excellent filtering effect by graph theory. Nevertheless, there are no related works to study its application for COVID-19 diagnosis. Therefore, we propose a novel COVID-19 graph in GCN, which considers the image differences between different equipment types and hospitals. Specifically, we first employ the popular 3D-CNN structure to extract image features from 3D-CT images. In this process, a transfer learning method based on predicting equipment type is used to initialize the parameters of 3D-CNN. After this process, every subject on the graph is represented by a feature vector. Besides, every subject gets an initial diagnosis score from 3D-CNN. Second, we design a COVID-19 graph in GCN to consider the differences between different equipment types, hospital information, and disease statuses of those training samples. We also combine the extracted features and the scores from 3D-CNN to construct edge weights. Third, we input our COVID-19 graph into a GCN model for the final diagnosis.

Overall, we apply a 3D-CNN to extract image features from CT images and then design a COVID-19 graph in GCN to complete the diagnosis. The main contributions are described as follows:

(1) We propose a transfer learning method by predicting equipment type to initialize the parameters of the 3D-CNN structure for extracting features from CT images.
(2) We design a COVID-19 graph in GCN, which considers the information of equipment type, hospital, and disease status. We compute edge weights based on the correlation distance of extracted features and the score differences of subjects from the 3D-CNN structure.
(3) We analyze the feature differences between different equipment types. Experimental results show that our method has a good diagnosis performance.

The rest of the paper is organized as follows. The related works of 3D-CNN and GCN are introduced in section Related Works. In section Methodology, our methodology is presented. The results of our experiments and analysis of feature differences between different equipment types are given in section Experiments and Results. Finally, this paper is concluded.

## Related Works

### 3D-CNN for Feature Extraction

As deep CNN can filter noise and reduce parameters, it is widely studied. The most popular CNN methods include LeNet-5, AlexNet, VGG-16, Inception-v1, ResNet-50, Inception-ResNet, and so on, which are successfully applied in semantic segmentation (18), object detection (19, 20), and image recognition and segmentation (21).

As CNNs usually contain numerous parameters to achieve good ACC, it is necessary to explore simple and efficient network architectures, especially for our time series CT scan images, which can be regarded as 3D images. Multitask learning incorporates the benefits from several related tasks to excavate features better. It can take the underlying common information that may be ignored by single-task learning. Eventually, it improves the performance, the robustness and stability of disease detection, or image segmentation (22, 23). Transfer learning can be used to improve a learner from one domain by transferring information from a related domain (24) and is widely used to initialize the parameters of a system (25–27). As the equipment information is usually acquired and is an essential feature to images, we propose to utilize the task of predicting equipment type to initialize the parameters of the 3D-CNN structure and finally employ it to improve the extracted features.

### GCN

A graph neural network (GNN) was proposed in 2009 (28), which is based on the graph theory (29), building the foundation of all kinds of graph networks (30–33). As one of the most famous graph networks, GCN mainly applies the convolution of Fourier transform and Taylor's expansion formula to improve filtering performance (34). With its excellent performance, GCN has been widely used in disease classification (34–38).

For graph theory, a node on the graph represents a subject's imaging data, and the edges establish interactions between each pair of nodes. Sarah et al. (35) integrated similarities between imaging information and distances between phenotypic information (e.g., sex, equipment type, and age) into edges for the prediction of autism spectrum disorder and the conversion to Alzheimer's disease (AD). Zhang et al. (36) combined an adaptive pooling scheme and a multimodal mechanism to classify Parkinson's disease (PD) status. Kazi et al. (37) designed different kernel sizes in spectral convolution to learn cluster-specific features for predicting mild cognitive impairment and AD. All these studies validate the effectiveness of GCN and show that the convolution operation is the key to prediction performance. On a graph, edges and edge weights determine the convolution operation. According to the characteristic of COVID-19 datasets, we design a COVID-19 graph to establish edges by considering equipment types, hospitals, and disease statuses. Current edge weights are roughly computed based on the correlation distance between image feature vectors, which is inaccurate and may affect the convolution performance. Therefore, we propose a combination mechanism, which combines the correlation distance of extracted features and the scores from 3D-CNN, to better compute the edge weights.

## Methodology

**Figure 1:**
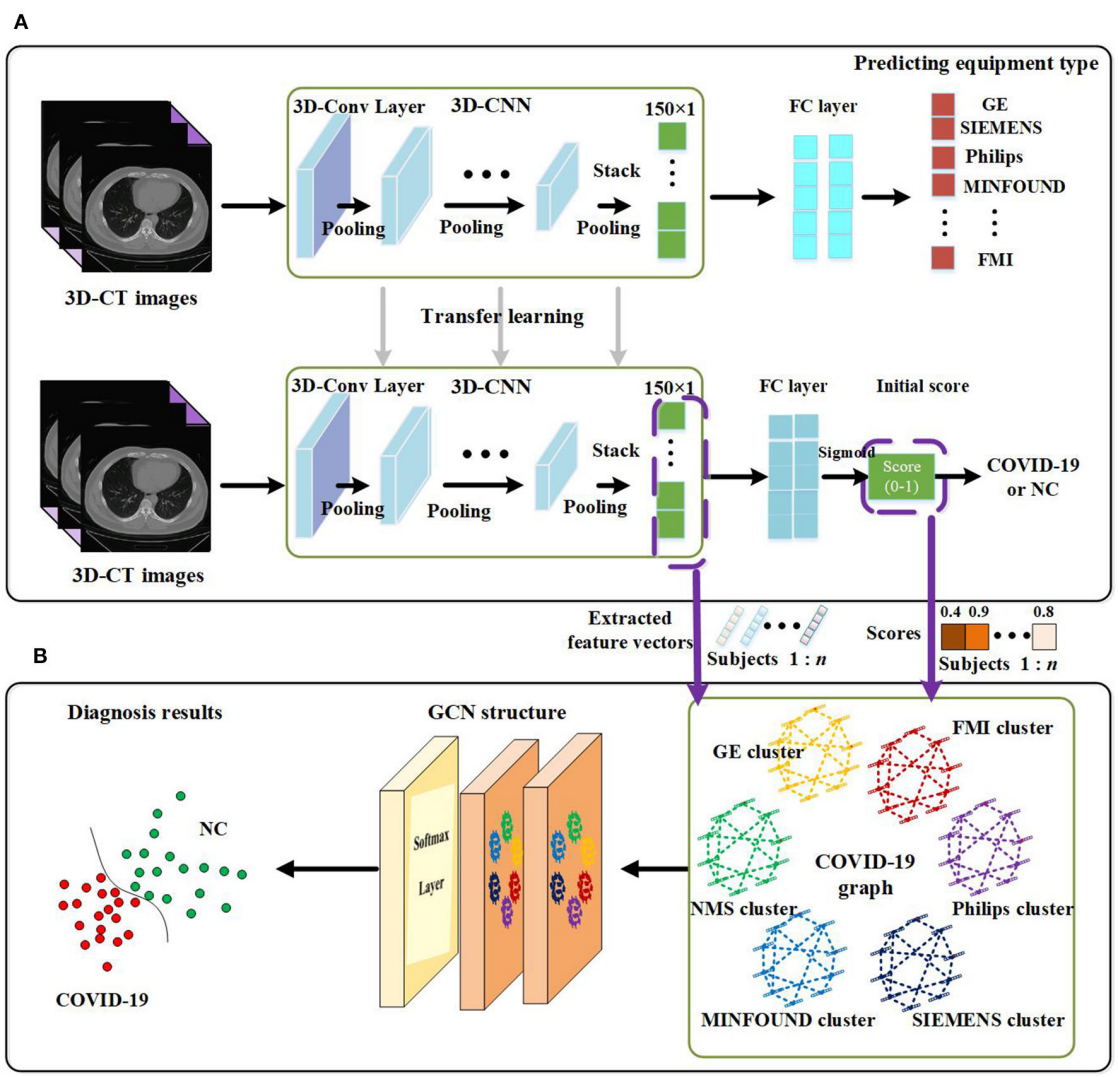
Overview of the proposed coronavirus disease 2019 (COVID-19) diagnosis framework. **(A)** Feature extraction. **(B)** GCN structure for final diagnosis.

The proposed method in this paper consists of two key parts. Using 3D-CNN, we extract image features from 3D-CT images and get an initial score for every subject. By designing our COVID-19 graph in GCN, we accomplish the COVID-19 diagnosis task. We first introduce the proposed 3D-CNN framework for feature extraction. Then we present the proposed COVID-19 graph and GCN. The overview of the proposed diagnosis mechanism is shown in Figure 1.

### Feature Extraction by Using 3D-CNN

**Figure 2:**
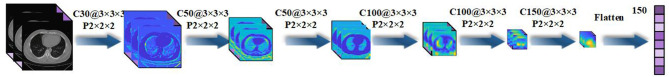
Details of the three-dimensional convolutional neural network (3D-CNN) structure for feature extraction.

The paper applies a 3D-CNN network (39, 40) to extract features from 3D-CT images. We first use z-score standardization to process the initial CT scans. Since the acquired datasets are not uniform and the 3D-CT images are of different sizes, we converted all the 3D-CT images into the same size of 64 × 64 × 32. Specifically, the 3D-CNN model has six convolutional layers and six max-pooling layers with a rectified linear unit (ReLU) as its activation function. The details of the 3D-CNN structure are shown in Figure 2. In the first layer, C30@3 × 3 × 3 denotes there are 30 convolution kernels and the kernel size is 3 × 3 × 3. P2 × 2 × 2 denotes the size of the pooling layer is 2 × 2 × 2.

Transfer learning is a widely used machine learning technique especially for comparatively little data in many fields (41, 42), which enables scientists to benefit from the knowledge gained from a previously trained model for a related task. Specifically, by applying transfer learning, we can exploit the valuable information that has been learned in one task to improve generalization in another. The popularly used machine learning transfers the weights that a network has learned at “task A” to a new “task B.”

As there are more than millions of parameters in our 3D-CNN and <1,000 samples for training 3D-CNN, it has great significance to applying transfer learning on our COVID-19 diagnosis task. In view of the fact the equipment type is an important factor that affects acquired images and is easy to acquire, we design a transfer learning method to transfer the weights of 3D-CNN based on the known equipment type, as shown in Figure 1. There are two tasks, including predicting equipment type and diagnosing COVID-19. We first employ 3D-CT images and their corresponding equipment type labels to train the first system of predicting equipment type. Then we transfer the weights of the 3D-CNN in the first system to the second system of diagnosing COVID-19. Finally, we adopt 3D-CT images and their corresponding COVID-19 labels in a training set to train the COVID-19 diagnosis system.

After the above processes, we get a well-trained COVID-19 diagnosis system by using transfer learning. Further, we utilize the trained COVID-19 diagnosis system to score every subject and extract all samples' features from the 3D-CNN structure. As shown in Figure 1, we finally get a 150 × 1 feature vector and a score value for every subject. Every extracted feature vector composes a node on the graph in GCN, and score values are used to establish edges between nodes. High-dimensional feature vectors will increase the burden on the following GCN, and we use recursive feature elimination (RFE) (43) to select features from the 150 × 1 feature vector to reduce the feature vector's dimensions. In detail, given an estimator (e.g., ridge classifier) that assigns weights to features, RFE selects features by recursively considering smaller and smaller sets of features. First, the estimator is trained on the initial set of features, and the importance of each feature is obtained. Then the least important features are pruned from the current set of features. This procedure is recursively repeated on the pruned set until the desired number of features to be selected is eventually reached (44–46).

### GCN

**Figure 3:**
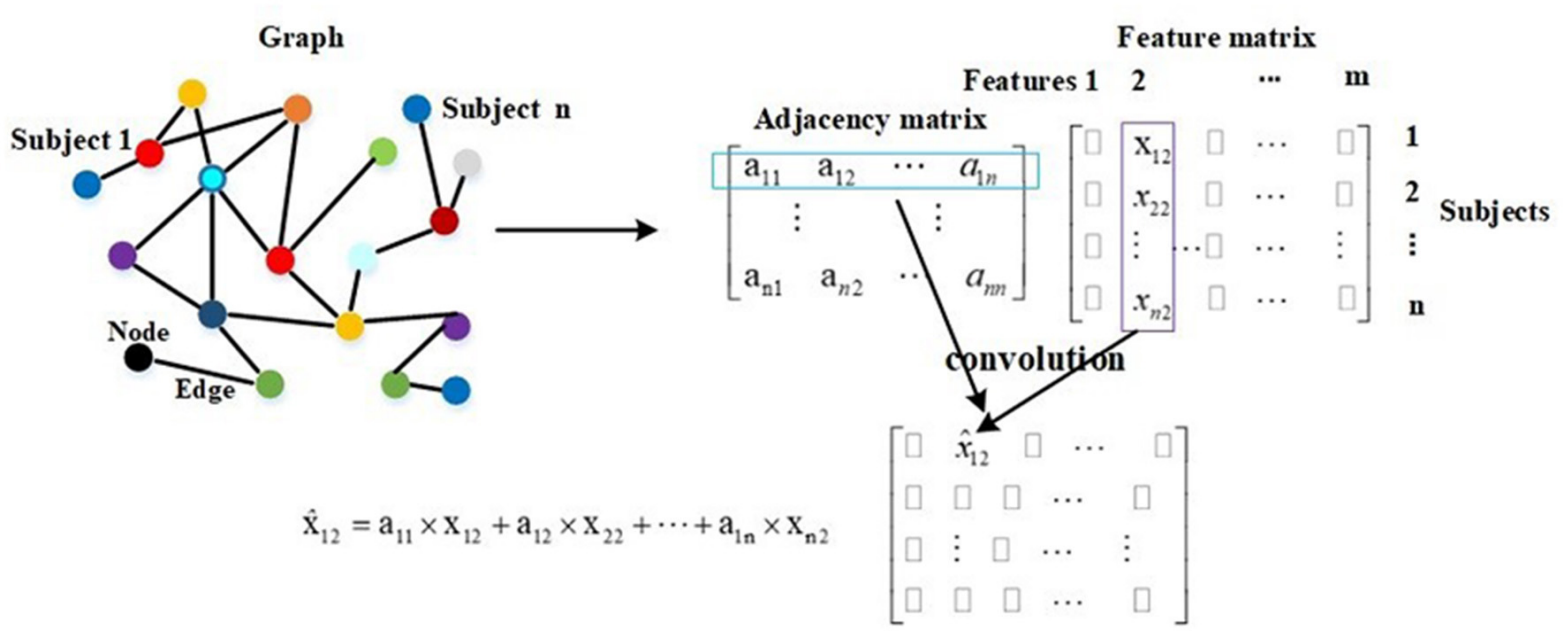
The description of graph theory.

Compared with traditional neural networks, GCN makes use of graph theory to improve performance, and the graph in GCN plays the role of filtering noise. On a graph, a node represents the feature vector of a subject, and an edge denotes the interaction between corresponding pair nodes. Graph theory takes all nodes on the graph to perform convolution, and edge weights are the key to the performance as they are the corresponding convolution coefficients. Thereby, they attract much attention (47, 48). The description of graph theory is shown in Figure 3. As shown, a node represents a subject, and there are total of *n* subjects, and everyone is represented by a 1 × *m* feature vector. In mathematical form, a graph with *n* nodes can be described as an *n* × *m* feature matrix pre-multiplied with an *n* × *n* adjacency matrix, where the adjacency matrix is composed of all edge weights. The *n* × *n* adjacency matrix plays the role of filtering noise. For example, for subject 1, the filtered feature 2 is computed as ${\hat{x}}_{12}=\overset{i=n}{\underset{i=1}{\sum }}a_{1i}\times x_{i2}$. The convolution coefficients determine the filtering effect, and improving them is our main contribution in this paper. There are usually millions of parameters in 3D-CNN, whereas only thousands of COVID-19 infected and non-infected subjects in the training process, and the insufficiency of samples introduces noise in extracted features, which further deteriorates the final diagnosis performance. The existing noise is given in **Figure 6**.

In this subsection, we propose a COVID-19 graph in GCN to establish edges to fit in the characteristics of the diagnosis task, which finally plays the role of improving the adjacency matrix.

#### COVID-19 Graph

We first consider the differences in the equipment types in our datasets, so we divide all nodes on the COVID-19 graph into several clusters. Those subjects with the same equipment type compose a cluster, which means the number of clusters matches the number of equipment types in our datasets. There are six clusters, including SIEMENS cluster, Philips cluster, NMS cluster, Minfound cluster, FMI cluster, and GE cluster. Every cluster corresponds to one equipment type. We do not establish the connections between nodes in different clusters, which can also be regarded as zero-weight edges. For nodes in the same cluster, we propose a novel method to establish their edges, which considers the hospital information and disease status of those training samples. On our COVID-19 graph, the proposed edge weights between two subjects in the same cluster are calculated as follows:

(1) $$\begin{array}{r} A\left(v,u\right)=Sim\left(F_{v},F_{u}\right)\times \left(1+r_{H}\left(H_{v},H_{u}\right){+r}_{s}\left(S_{v},S_{u}\right)\right) \end{array}$$

(2) $$r_{H}\left(H_{v},H_{u}\right)=\{\begin{array}{c} 1,\;H_{v}=H_{u}\;\\ 0,\;H_{v}\neq H_{u}\; \end{array}$$

(3) $$r_{S}\left(S_{v},S_{u}\right)=\{\begin{array}{c} 1,\;S_{v}=S_{u}\;\\ 0,\;S_{v}\neq S_{u}\;\\ 0,\;S_{v}\;or\;S_{u}\;is\;unknown\; \end{array}$$

where all edge weights compose adjacency matrix ***A***. ***A***(*v, u*) is the edge weight between subject *v* and subject *u*, Sim(·) denotes the similarity of imaging information, ***F***_*v*_ and ***F***_*u*_ represent the feature vectors of subject *v* and subject *u*, respectively. *r*_*H*_ represents the distance between hospitals, *r*_*S*_ represents the distance of disease status (the statuses of those training samples on the graph are known), *S*_*v*_ and *S*_*u*_ are the subjects' disease statuses (COVID-19 infected case or healthy case), and *H*_*v*_ and *H*_*u*_ denote their corresponding hospital. For example, if the images of subject *v* and subject *u* are acquired from the same hospital, we set *H*_*v*_ = *H*_*u*_. If subject *v* and subject *u* are all COVID-19 infected cases (or healthy cases), we set *S*_*v*_ = *S*_*u*_.

##### Edge Weights Based on Correlation Distance

The popular method for evaluating the similarity of imaging information is based on the correlation distance as follows (35):

(4) $$\begin{array}{r} \text{Sim}\left(F_{v},F_{u}\right)=\exp\left(-\frac{{\left[\rho \left(F_{v},F_{u}\right)\right]}^{2}}{2\sigma^{2}}\right) \end{array}$$

where ρ(·) is the correlation distance function and σ is the width of the kernel. We compute the correlation distances between each pair of nodes, and the σ is set as the mean value of the correlation distances according to the work in Zhang et al. (36).

By Equations (1)–(4), we can get an adjacency matrix ***A***_*f*_, which represents the adjacency matrix constructed based on the correlation distance of extracted feature vectors.

##### Edge Weights Based on Scores

Using correlation distance to compute similarities as in Equation (4) is rough and deteriorates convolution performance to some extent. We propose a method to compute the similarities in view that the 3D-CNN has good capability to excavate in-depth features. Employing 3D-CNN to extract features from CT images, we also get a diagnosis score for every subject, as shown in Figure 1. Based on these scores, the proposed similarities are calculated as follows:

(5) $$\begin{array}{r} \text{Sim}\left(F_{v},F_{u}\right)=\exp\left(-\frac{{\left[{Score}_{v}-{Score}_{u}\right]}^{2}}{2\sigma^{2}}\right) \end{array}$$

where *Score*_*v*_ and *Score*_*u*_ denote the scores of subject *v* and subject *u* from 3D-CNN diagnosis system, respectively, and σ is also the width of the kernel and is set as the mean value of the correlation distances according to the work in Zhang et al. (36). Based on Equations (1)–(3), (5), we can also get an adjacency matrix ***A***_*s*_.

##### Combined Adjacency Matrix

After getting an adjacency matrix ***A***_*f*_ based on correlation distance and an adjacency matrix ***A***_*s*_ based on initial scores, we further design a method to combine the two adjacency matrices to get a robust adjacency matrix. The combined adjacency matrix ***A***_*c*_ is calculated as follows:

(6) $$\begin{array}{r} A_{c}={a\times A}_{f}+b\times A_{s} \end{array}$$

where *a* and *b* are corresponding coefficients, and we set the two coefficients as 0.5 in this paper.

Then we can form our COVID-19 graph, which includes the extracted features from 3D-CT images and the edges represented by the combined adjacency matrix.

#### Spectral Theory and GCN Structure

In GCN series methods, the adjacency matrix is processed to achieve a better filtering effect and computational efficiency (49). The spectral convolution can be described as the multiplication of a signal ***x*** ∈ ℝ^*n*^ (a scalar for every node) with a filter *g*_***θ***_ = diag (***θ***) by

(7) $$\begin{array}{r} g_{\theta }*x=Ug_{\theta }\left(\Lambda \right)U^{T}x=\overset{K}{\underset{k=0}{\sum }}\theta_{k}T_{k}\left(\tilde{L}\right)x \end{array}$$

where ***U*** is the matrix of eigenvectors and is computed from the formula ***L*** = ***I***_*N*_ – ***D***^−(1/2)^***A***_ac_***D***^−(1/2)^ = ***U*Λ*U***^*T*^. ***I***_*N*_ is the identity matrix, and ***D*** represents the diagonal degree matrix. *g*_***θ***_(**Λ**) is well-approximated by a truncated expansion in terms of Chebyshev polynomials to the *K*th order. θ_*k*_ is a vector of Chebyshev coefficients, and *T*_*k*_ is the Chebyshev polynomial function, $\tilde{L}=\frac{2}{\lambda_{\max}\Lambda -\;I_{N}}$.

After the above spectral convolution is applied, combined adjacency matrix ***A***_c_ is approximated by $\overset{K}{\underset{k=0}{\sum }}\theta_{k}T_{k}\left(\tilde{L}\right)$. When polynomial order *K* is adjusted, a different filtering effect can be obtained. It is worth mentioning that the adjacency matrix is computed according to Equations (1)–(7).

As shown in Figure 1, there are two graph convolutional layers with a ReLU function as the activation function and one softmax function as the final output layer. Let $\hat{A}=\overset{K}{\underset{k=0}{\sum }}\theta_{k}T_{k}\left(\tilde{L}\right)$, and the formula of the two-layer GCN is as follows:

(8) $$\begin{array}{r} Z=\text{softmax}\left(\hat{A}ReLU\left(\hat{A}XW^{\left(0\right)}\right)W^{\left(1\right)}\right) \end{array}$$

The GCN model is trained and tested using the whole graph as input. Let ***F***_*i*_ and ***F***_*j*_ represent the feature vectors of subject *i* and subject *j*, respectively. ***X*** = [***F***_1_; ***F***_2_; …; ***F***_*n*_] represents the feature vectors of all subjects. In the training and test processes, the feature vector ***F***_*i*_ is approximated by $\overset{j=n}{\underset{j=1}{\sum }}{\hat{A}}_{i,j}F_{j}$ in every layer, and this is described by $\hat{A}X$. It is also embodied in Figure 3. In Equation (8), ***Z*** represents the labels of training and test samples. In the training process, the input is ***X***, the feature vector of every training sample is approximated by $\overset{j=n}{\underset{j=1}{\sum }}{\hat{A}}_{i,j}F_{j}$ in every layer, and the labels of test samples in ***Z*** are masked. The training samples and their labels are used for training GCN. In the test process, the input is also ***X***, the feature vector of every test sample is approximated by $\overset{j=n}{\underset{j=1}{\sum }}{\hat{A}}_{i,j}F_{j}$ in every layer, and after a two-layer trained GCN, we get their prediction results. In other words, in the training process, the feature vectors of test samples are used to help update the feature vectors of training samples. In the test process, the feature vectors of training samples are also used to help update the feature vectors of test samples. This is the meaning of graph theory for classification, and this is presented in Figure 3 and Equation (8). In adjacency matrix $\hat{A}$, every row of elements corresponds to the convolution coefficients (convolution kernel) of a subject. For example, the *i* row of elements in $\hat{A}$ represents the convolution coefficients of subject *i* as shown in Figure 3.

## Experiments and Results

The proposed methodology is implemented on a database of CT scan images from open sources (50–53). The dataset contains 399 COVID-19 infected cases and 400 normal controls with six equipment types. The equipment type information is shown in Table 1.

**Table 1:** The equipment type information in the dataset.

**Equipment type**	**Number**
SIEMENS	176
Philips	108
NMS	154
FMI	160
MINFOUND	100
GE	101

The parameters of the proposed GCN structure are as follows: learning rate is 0.005, dropout rate is 0.1, *l*_2_ regularization is 5 × 10^−4^, the number of epochs is 200, the number of neurons per layer is 64, and the number of extracted features is 20. Classification ACC, SEN, specificity (SPE), and area under receive operation curve (AUC) are selected as evaluation metrics.

This section is divided into seven parts. First, we evaluate the performance of the proposed transfer learning method on the 3D-CNN structure. Second, we test the performance of our GCN structure by comparing it with traditional methods. Third, we present the effect of different equipment types on extracted features. Fourth, we analyze the influence of the number of extracted features. Fifth, we analyze the effect of the width of kernel. Sixth, we analyze the performance of the GCN method on other public datasets. Last, we compare our methods with related works.

### Performance of the Proposed Transfer Learning Method

In the proposed feature extraction framework, the task of predicting equipment type is utilized to initialize the parameters of the 3D-CNN structure, as shown in Figure 1. The transfer learning method improves the performance of 3D-CNN, and Table 2 shows the method's effectiveness on the diagnosis performance of the 3D-CNN diagnosis framework.

**Table 2:** Performance of the 3D-CNN, 3D-ResNet18, and PENet diagnosis frameworks with and without our transfer learning method (5-fold cross validation).

**Model**	**ACC (%)**	**SEN (%)**	**SPE (%)**	**AUC (%)**
3D-CNN	94.3 ± 0.8	90.1 ± 1.1	98.7 ± 0.5	98.7 ± 0.03
3D-ResNet18	93.1 ± 1.4	93.9 ± 1.9	92.4 ± 1.2	97.9 ± 0.04
PENet	94.5 ± 1.2	90.7 ± 1.5	97.7 ± 0.7	98.2 ± 0.01
3D-CNN + transfer learning	95.6 ± 1.0	96.2 ± 1.6	95.1 ± 0.6	99.6 ± 0.02
3D-ResNet18 + transfer learning	94.9 ± 1.5	95 ± 1.8	98 ± 0.9	99.4 ± 0.03
PENet + transfer learning	95.2 ± 1.8	98.4 ± 2.1	96.2 ± 1.4	99.9 ± 0.03

As shown in Table 2, with our transfer learning method, the ACC of the 3D-CNN diagnosis framework increases by 1.3%, SEN increases by 6.1%, and AUC increases by 1.1%, whereas SPE decreases by 3.6%. The ACC of the 3D-CNN diagnosis framework reaches 95.6%. These improvements support us getting better extracted features with our transfer learning method. In addition, we also test our transfer learning method on 3D-ResNet18 and PENet (54). Table 2 shows that our transfer learning method can improve ACC by 1.8 and 0.7%. Compared to 3D-CNN, PENet has a little effect on performance improvement whereas 3D-ResNet18 deteriorates performance, and they consume much more time. In our paper, we use the simplest 3D-CNN structure to extract features as its stable performance.

### Performance of Our GCN Diagnosis Framework

**Figure 4:**
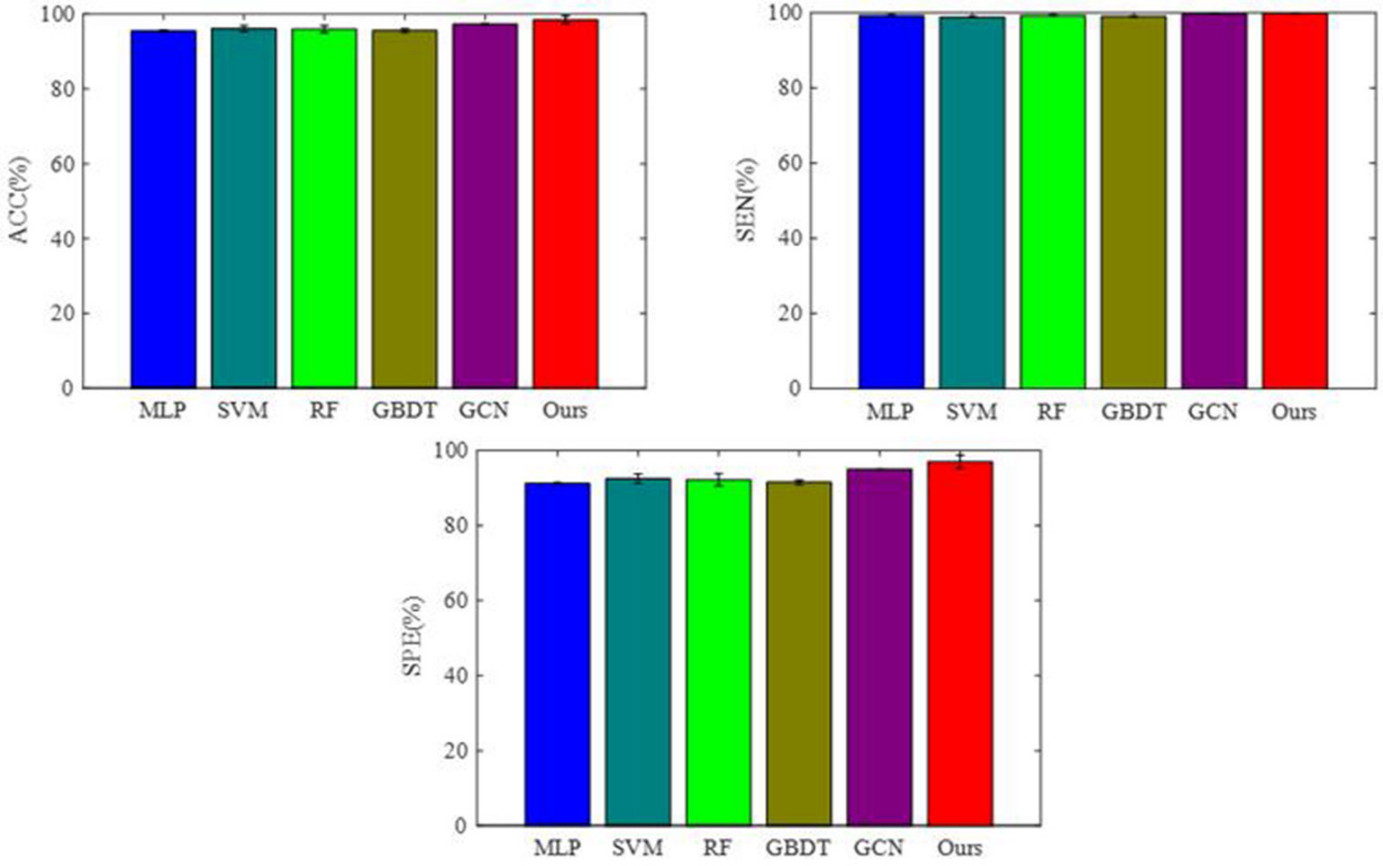
Performance of the different classifiers based on the extracted features.

**Figure 5:**
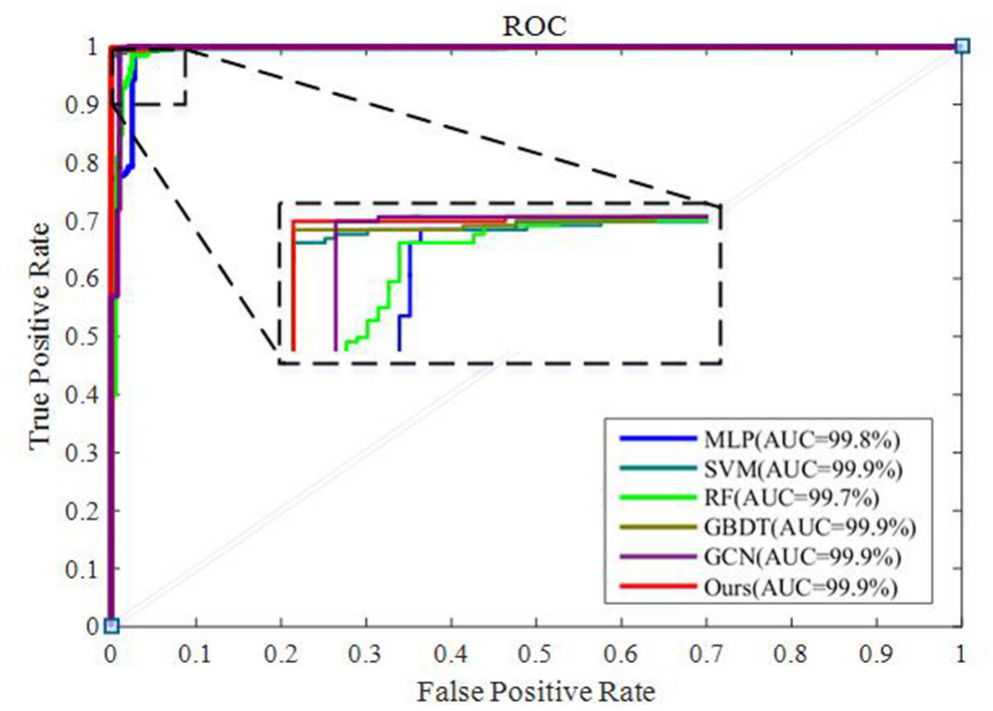
ROC curves of the different classifiers based on the extracted features.

To integrate equipment type information, hospital information, and disease status information, we design our GCN structure to accomplish the diagnosis task based on the extracted features and initial scores from the 3D-CNN framework. In this subsection, we compare our novel GCN structure with some other classifiers based on the extracted features. The compared classifiers include multiple layer perception (MLP), support vector machine (SVM), random forest (RF), gradient boosting decision tree (GBDT), and traditional GCN (35). The results of the performance comparison are shown in Table 3. Figure 4 shows the performance comparison by histograms, and it shows our GCN has better performance than others. Figure 5 describes the ROC curves of different methods, and it shows that our GCN has better AUC values.

**Table 3:** Diagnosis performance of different classifiers based on the extracted features (5-fold cross validation).

**Model**	**ACC (%)**	**SEN (%)**	**SPE (%)**	**AUC (%)**
MLP	95.5 ± 0.27	99.3 ± 0.05	91.2 ± 0.2	99.8 ± 0.01
SVM	96.1 ± 0.81	98.9 ± 0.05	92.5 ± 1.25	99.9 ± 0.01
RF	96 ± 0.94	99.3 ± 005	92.2 ± 1.62	99.7 ± 0.02
GBDT	95.6 ± 0.44	99.1 ± 0.09	91.5 ± 0.55	99.9 ± 0.01
GCN	97.3 ± 0.27	99.7 ± 0.05	95 ± 0.1	99.9 ± 0.02
Ours	98.5 ± 0.96	99.9 ± 0.04	97 ± 1.73	99.9 ± 0.01

As shown in Table 3, compared to the diagnosis performance of the 3D-CNN framework with transfer learning in Table 2, there is virtually no performance improvement by using the 3D-CNN extracted features and then using traditional classifiers (MLP, SVM, RF, and GBDT) for the final diagnosis. The traditional GCN (35) has slight performance improvement, with mean ACC increasing by 1.7% and mean SEN increasing by 3.5%. Nonetheless, with our proposed COVID-19 graph in GCN, mean ACC increases by 2.9%, mean SEN increases by 3.7%, and mean SPE increases by 1.9%. These results show that our COVID-19 graph can improve performance significantly. In short, with our COVID-19 graph in GCN, the performance of GCN gets significant improvement, with final mean ACC, SEN, SPE, and AUC reaching 98.5, 99.9, 97, and 99.9%.

### Effect of Equipment Type on Extracted Features and the Filtering Effect of Our Graph on Features

**Figure 6:**
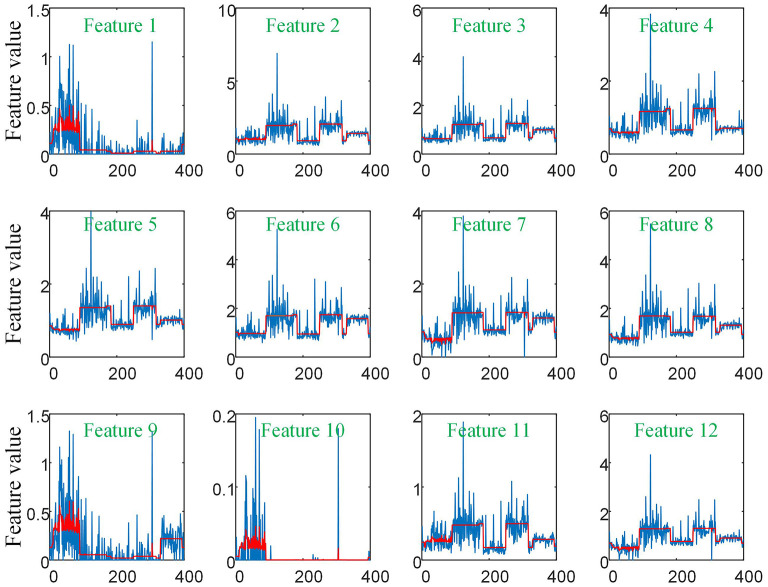
The filtering effect of the different-equipment-type graph on the extracted features by comparing ***X*** with $\hat{A}X$. ***X*** is the original feature matrix of all equipment types' subjects, which is represented by the blue line, and $\hat{A}X$ represents the filtered features by pre-multiplying with the adjacency matrix $\hat{A}$, which is represented by the red line.

**Figure 7:**
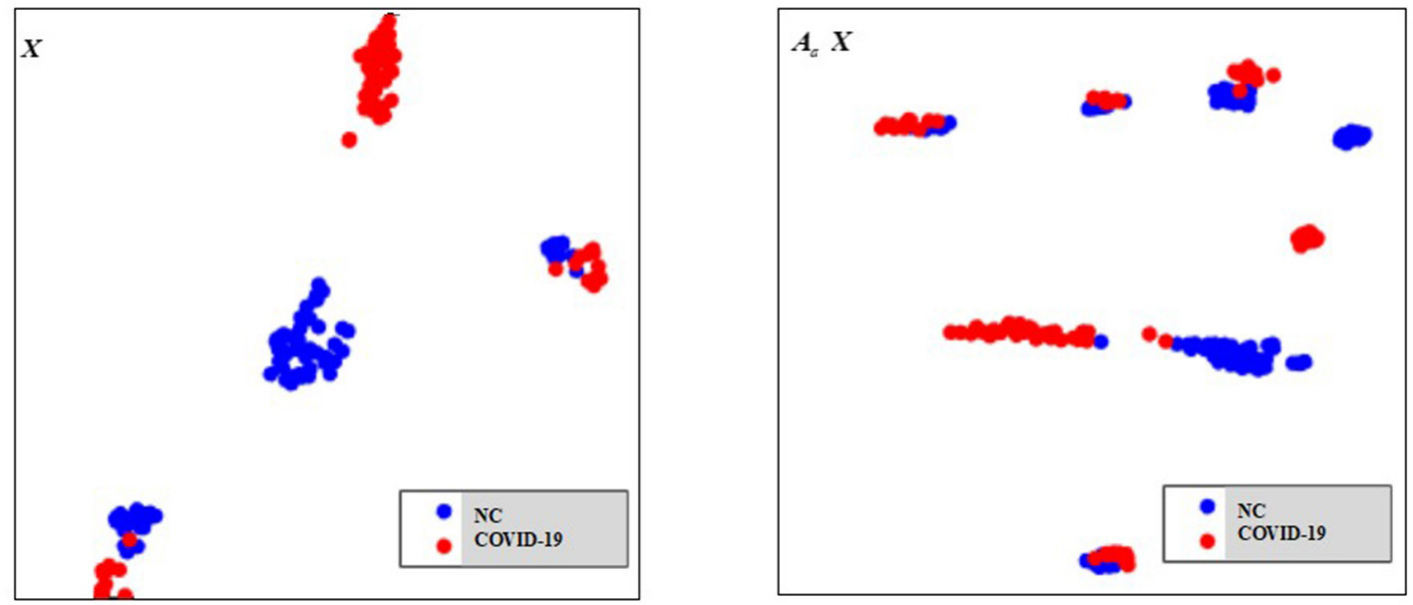
The t-SNE visualization results of feature maps, where we compare ***X*** with $\hat{A}X$.

As there are no related works to evaluate the filtering effect of GCN series methods for disease prediction, we propose to describe it by comparing ***X*** with $\hat{A}X$. ***X*** represents the feature matrix that is composed by all subjects' feature vectors, $\hat{A}$ is our adjacency matrix, and $\hat{A}X$ represents the feature matrix after filtering. As there are no real feature values, we propose to use mean values to represent real feature values and use standard deviation to describe noise level in this subsection. Figure 6 visualizes the filtering effect of the different-equipment-type graph on the extracted features by comparing ***X*** with $\hat{A}X$, and the detailed effect on mean and standard deviation is given in Table 4. Figure 7 shows t-SNE visualization results of feature maps where we compare ***X*** with $\hat{A}X$.

**Table 4:** The mean values and standard deviations of the top nine most discriminative features in our six clusters.

**Top 9 features**		**SIEMENS, mean ± SD**	**Philips, mean ± SD**	**NMS, mean ± SD**	**FMI, mean ± SD**	**MINFOUND, mean ± SD**	**GE, mean ± SD**
Feature 1	*X*	1.03 ± 0.322	1.03 ± 0.322	1.96 ± 0.863	1.48 ± 0.702	1.42 ± 0.123	0.93 ± 0.234
	$\hat{A}X$	1.03 ± 0.002	1.02 ± 0.053	1.96 ± 0.003	1.48 ± 0.574	1.42 ± 0.001	0.93 ± 0.002
Feature 2	*X*	0.69 ± 0.163	0.64 ± 0.154	1.22 ± 0.481	0.96 ± 0.378	1.00 ± 0.084	0.66 ± 0.146
	$\hat{A}X$	0.69 ± 0.001	0.63 ± 0.002	1.22 ± 0.002	0.96 ± 0.304	1.00 ± 0.002	0.66 ± 0.001
Feature 3	*X*	0.69 ± 0.155	0.59 ± 0.166	1.16 ± 0.452	0.95 ± 0.372	0.71 ± 0.086	0.70 ± 0.153
	$\hat{A}X$	0.69 ± 0.001	0.60 ± 0.013	1.16 ± 0.001	0.96 ± 0.297	0.71 ± 0.001	0.70 ± 0.001
Feature 4	*X*	0.85 ± 0.166	0.75 ± 0.154	1.35 ± 0.453	1.14 ± 0.331	1.01 ± 0.082	0.89 ± 0.122
	$\hat{A}X$	0.86 ± 0.001	0.75 ± 0.024	1.36 ± 0.001	1.15 ± 0.259	1.01 ± 0.001	0.89 ± 0.001
Feature 5	*X*	1.02 ± 0.263	0.97 ± 0.222	1.70 ± 0.637	1.34 ± 0.512	1.58 ± 0.123	0.96 ± 0.175
	$\hat{A}X$	1.02 ± 0.002	0.97 ± 0.015	1.70 ± 0.002	1.34 ± 0.404	1.58 ± 0.002	0.96 ± 0.001
Feature 6	*X*	0.69 ± 0.227	0.48 ± 0.217	1.21 ± 0.466	0.98 ± 0.328	1.07 ± 0.104	0.68 ± 0.137
	$\hat{A}X$	0.69 ± 0.003	0.48 ± 0.053	1.21 ± 0.003	0.98 ± 0.247	1.07 ± 0.002	0.68 ± 0.001
Feature 7	*X*	0.93 ± 0.286	0.78 ± 0.204	1.69 ± 0.663	1.34 ± 0.449	1.31 ± 0.101	0.93 ± 0.158
	$\hat{A}X$	0.93 ± 0.003	0.78 ± 0.035	1.69 ± 0.001	1.35 ± 0.334	1.31 ± 0.003	0.93 ± 0.002
Feature 8	*X*	0.24 ± 0.103	0.37 ± 0.332	0.48 ± 0.255	0.33 ± 0.213	0.28 ± 0.042	0.19 ± 0.064
	$\hat{A}X$	0.24 ± 0.001	0.36 ± 0.091	0.48 ± 0.001	0.33 ± 0.162	0.28 ± 0.001	0.19 ± 0.002
Feature 9	*X*	0.69 ± 0.221	0.49 ± 0.227	1.28 ± 0.535	1.03 ± 0.371	0.90 ± 0.103	0.70 ± 0.131
	$\hat{A}X$	0.69 ± 0.002	0.50 ± 0.053	1.28 ± 0.001	1.03 ± 0.275	0.90 ± 0.003	0.70 ± 0.003

*We compare features' mean values and standard deviations with or without pre-multiplication with the adjacency matrix $\hat{A}$*.

As shown in Figure 6 and Table 4, there is much difference in the mean values of the same feature between different clusters. For example, the mean values of feature 1 with ***X*** in our six clusters are 1.03, 1.03, 1.96, 1.48, 1.42, and 0.93. The mean values of feature 2 with ***X*** in our six clusters are 0.69, 0.64, 1.22, 0.96, 1.00, and 0.66. These results validate that there are differences in images between different equipment types. Based on the big differences, we design our COVID-19 graph to divide all samples into several clusters (a cluster includes those samples acquired by one kind of equipment type) and establish edge connections between those samples from the same cluster. By pre-multiplying with adjacency matrix $\hat{A}$, the noise in extracted features is well-suppressed, as shown in Figure 6, where red lines have a small fluctuation and blue lines have a big fluctuation.

In Table 4, it is also shown that there are different noise levels between different features in the same cluster and also different noise levels between the same features in different clusters. For example, for feature 1, the standard deviations of our six clusters with ***X*** are 0.32, 0.32, 0.86, 0.70, 0.12, and 0.23. For feature 2, the standard deviations of our six clusters with ***X*** are 0.16, 0.15, 0.48, 0.37, 0.08, and 0.14. These results show that there is much noise on the extracted features in the NMS cluster and the FMI cluster. Furthermore, the comparison of the mean values and standard deviations of $\hat{A}X$ with those of ***X*** shows that mean values were kept stable, whereas standard deviations reduced significantly. For example, for all features, the standard deviations of four clusters (i.e., SIEMENS, NMS, MINFOUND, and GE) are very small. These results show that the GCN has an excellent filtering effect.

### Effect of the Number of Extracted Features

**Figure 8:**
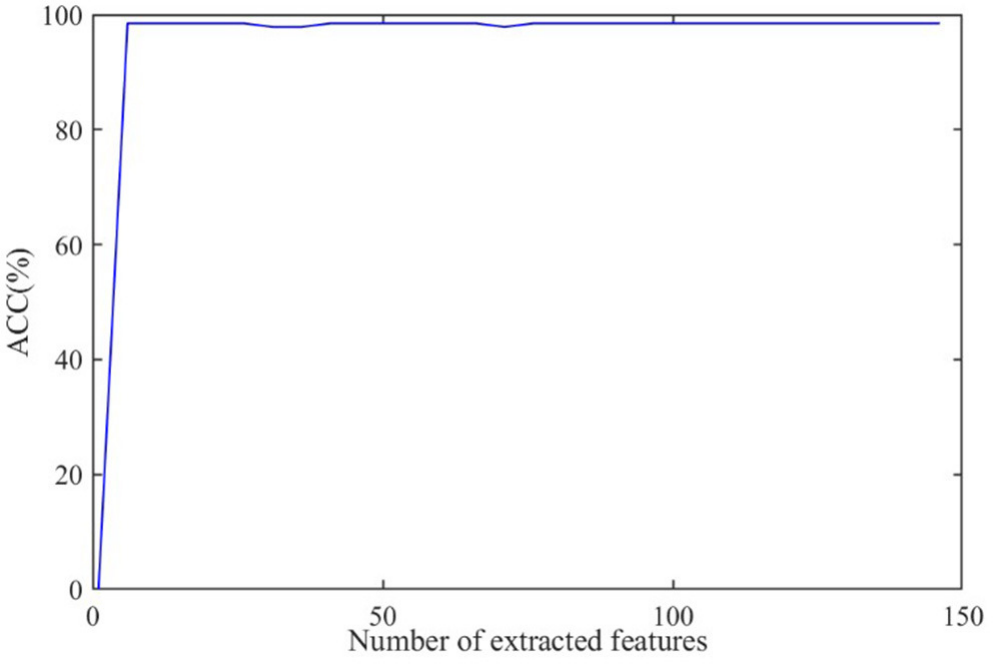
The effect of the number of extracted features on diagnosis accuracy.

The discriminative features are extracted from CT images by using 3D-CNN and initially form a 150 × 1 feature vector for every subject. Further, we apply RFE to select the principal features. The effect of the number of final selected features on diagnosis ACC is shown in Figure 8, where the number of extracted features varies from 0 to 150.

As shown in Figure 8, the ACC value increases to a stable value rapidly and maintains stability after the number exceeds 10. As a large number can increase the burden on GCN, we set the number of the extracted features as 20 in this paper.

### Effect of the Width of Kernel

The width of kernel *K* in Equation (7) means the filter learned for neighbors *K* hops away from the node at the center of the receptive field, and it affects classification performance according to a previous study (35). We test the effect of *K* on performance in this subsection. Here, we test *K* ∈ {1, 2, 3, 4, 5, 6}. By setting different *K* values in our GCN method, we get their corresponding ACC as {97.9, 98.3, 98.5, 98.3, 98.1, 98.1%}.

As the above results shows, there are a few variations on ACC with different *K* values. Specifically, with *K* set as 3, the best ACC is reached, and this result is consistent with the work in Parisot et al. (35) and Ktena et al. (38). Therefore, we set *K* = 3 in our experiments.

### Performance of the GCN Method on Other Public Datasets

There are several large public datasets available, but no equipment type information is included. In this subsection, we further test the GCN method by combining other datasets. The experiment includes the dataset from http://ncov-ai.big.ac.cn/download?lang=en (55) and the dataset from https://mosmed.ai/datasets/covid19_1110 (56). Constrained by our computer memory, we selected a total of 1,560 COVID-19 cases and 1,560 NC cases randomly from the above datasets. As there is no equipment type information in the above datasets, we use 3D-CNN to extract features, ignoring the transfer learning method, and we treat all samples as one cluster in the COVID-19 graph. The experimental results are listed in Table 5.

**Table 5:** Diagnosis performance of the GCN method on the large dataset (5-fold cross validation).

**Model**	**ACC (%)**	**SEN (%)**	**SPE (%)**	**AUC (%)**
MLP	99.5 ± 0.12	99.1 ± 0.02	100 ± 0.1	99.5 ± 0.04
SVM	99.1 ± 0.31	99.3 ± 0.02	98.4 ± 0.7	99.6 ± 0.02
RF	99 ± 0.12	98.9 ± 0.03	99.1 ± 0.1	99.6 ± 0.02
GBDT	98.7 ± 0.11	97.1 ± 0.02	99.2 ± 0.2	99.4 ± 0.01
GCN	99.6 ± 0.17	100 ± 0.03	99.3 ± 0.1	99.8 ± 0.01
Ours	99.8 ± 0.36	99.6 ± 0.01	100 ± 0.5	100 ± 0.02

As shown in Table 5, with a large dataset, the performance of COVID-19 diagnosis is satisfactory with an ACC reaching 99.5% by using the MLP method. Compared to the ACC with the MLP method, the ACC by using our GCN method increases by 0.3%. Compared to the 1.7% ACC improvement in Table 3, the little performance improvement validates that our GCN method relatively adapts to the few-shot learning task.

### Comparison With Related Works

Table 6 shows the diagnosis performance of our method and related methods. It is observed that our method achieves the best ACC, with it reaching 98.5%. Compared to related works, our method improves the ACC by 2.6–13.6%. In terms of SEN and SPE, our method also shows the best performance.

**Table 6:** Algorithm comparison with the related works.

**References**	**Data type**	**Method**	**Database size**	**ACC (%)**	**SEN (%)**	**SPE (%)**
Sun et al. (9)	CT	Transfer learning, CNN	325 COVID-19, 740 others	89.5	87	88
Abbas et al. (57)	X-ray	Decompose, transfer, compose, CNN	105 COVID-19, 80 NC	95.12	97.91	91.87
Shi et al. (58)	CT	Infection size aware random forest	1,658 COVID-19, 1,027 CAP	87.9	90.7	83.3
Sethy and Behera (59)	X-ray	ResNet50, SVM	158 COVID-19, 158 others	95.38	97.29	93.47
		DenseNet201, SVM		93.88	94.35	93.41
		XceptionNet, SVM		93.91	94.76	93.06
Narin et al. (60)	X-ray	Inception-ResNetV2	50 COVID-19, 50 CAP	87	84	90
Jin et al. (61)	CT	CNN	497 COVID-19, 1,385 others	94.1	90.19	95.76
Butt et al. (62)	CT	ResNet18, location attention	219 COVID-19, 399 others	86.7	98.2	92.2
Bai et al. (63)	CT	EfficientNet-B4, two-layer fully connected NN	521 COVID-19, 655 others	96	95	96
Ours	CT	3D-CNN, transfer learning, GCN	399 COVID-19, 400 NC	98.5	99.9	97

### Discussion

Diagnosis of COVID-19 utilizing 3D-CT images is a few-shot learning task. Specifically, there are more than millions of parameters in our 3D-CNN and <1,000 samples for training 3D-CNN. Although we propose a transfer learning method by predicting equipment type to initialize the 3D-CNN parameters, which improves the performance to some extent, there are still much noise existing in the extracted features as shown in Figure 6. It is also shown that there is much difference on the same features between different clusters. This difference shows that equipment type has a big effect on images and supports the good performance of our transfer learning method. In view of the big difference, we propose our COVID-19 graph, which divides all samples into several clusters and samples with the same equipment type composing a cluster, to suppress the existing noise in extracted features. The purpose of our COVID-19 graph is to improve the filtering effect, and the filtering principle is shown in Figure 3. With the application of our COVID-19 graph, Figure 6 shows that the noise is well-suppressed, and this is the key for our performance improvement.

Our main contribution is analyzing the effect of equipment type on images. By analyzing its effect on the extracted features, we proposed a transfer learning method and a GCN classifier for COVID-19 diagnosis. It is worth mentioning that there are still some limitations. Our GCN method is limited to a binary classification task, where the more important and difficult challenge is discriminating COVID-19 from other abnormal cases (e.g., pneumonia) and normal controls, and we will study this issue in our future work. Our work relatively adapts to a few-shot learning task as the advantage of GCN lies in its filtering effect.

## Conclusions

In this study, we proposed a novel method based on 3D-CNN and GCN for COVID-19 diagnosis. The proposed method considers three pieces of information: the equipment type, hospital information, and the disease status of the training samples. Comparing the diagnosis results of our method with the results of using 3D-CNN for diagnosis shows that using GCN with the three pieces of information can result in a 4.2% improvement on ACC. The comparison result validates that the three pieces of information are essential to CT images and effective for ACC improvement. The excellent performance of using the task of predicting equipment type to initialize the parameters of 3D-CNN also validates that the equipment type is a key information of CT images. The analysis of the extracted features from 3D-CNN in different clusters shows diversified noise levels across different clusters. We can conclude that there exists disparate imaging quality with the use of different CT equipment. Finally, our method achieves excellent performance, with a diagnosis ACC reaching 98.5%.

## Data Availability Statement

The dataset used in this study is from the open sources and can be downloaded from the following links: https://github.com/ahmadhassan7/Covid-19-Datasets, https://www.medrxiv.org/, https://www.biorxiv.org/, https://www.kaggle.com/mohammadrahimzadeh/covidctset-a-large-covid19-ct-scans-dataset, and http://ncov-ai.big.ac.cn/download?lang=en, https://mosmed.ai/datasets/covid19_1110.

## Author Contributions

Conception, design, and statistical analysis were performed by XL and YZ. Drafting the manuscript and editing were performed by JW, QY, YL, and JT. All authors read and approved the final manuscript.

## Conflict of Interest

The authors declare that the research was conducted in the absence of any commercial or financial relationships that could be construed as a potential conflict of interest.
